# Factors determining recurrence in transient global amnesia

**DOI:** 10.1186/s12883-020-01658-8

**Published:** 2020-03-06

**Authors:** Rebecca Tynas, Peter K. Panegyres

**Affiliations:** 1 The University of Western Australia, Nedlands, Australia; 2 Neurodegenerative Disorders Research Pty Ltd, 4 Lawrence Avenue, West Perth, Western Australia 6005

**Keywords:** Transient global amnesia, Recurrent, Risk factors, Diffusion-weighted imaging, Dementia

## Abstract

**Background:**

Aetiology of transient global amnesia (TGA) remains uncertain, though many have been proposed, including ischaemic, migrainous or epileptic pathologies.

**Methods:**

We attempted to determine risk factors for TGA, as well as prognostic factors that may cause recurrence. We evaluated clinical history, family history and magnetic resonance diffusion-weighted imaging (DWI) studies of 93 prospective patients with TGA. Patients were followed from 2004 to 2016. Fifteen of 93 (16%) patients experienced a recurrence of TGA.

**Results:**

Among precipitating events, physical activities inducing Valsalva-like manoeuvres were most common, followed by emotional stress. Eighty-four patients had possible comorbidities or risk factors for TGA, though no single risk factor was ubiquitous. Risk factors associated with recurrence were head injury (isolated vs. recurrent, 16.7% vs. 53.5%, *p* &lt; 0.01), depression (isolated vs. recurrent, 15.4% vs 46.7%, *p* = 0.01) and family history of dementia (isolated vs. recurrent, 20.5% vs. 46.7%, *p* = 0.03). Of 15 patients with confirmed recurrent TGA, two developed dementia and four subjective memory impairment. DWI lesions were observed in 24 patients and were located anywhere within the hippocampus.

**Conclusions:**

DWI lesions were not significantly associated with outcomes (recurrence, subjective memory impairment, dementia). We have found that depression, previous head injury and family history of dementia may predict TGA recurrence.

## Background

Transient global amnesia (TGA) presents as sudden onset anterograde amnesia, with some features of retrograde amnesia, without residual cognitive impairment, of duration &lt; 24 h. Typically, it occurs in individuals aged 50–80 years, with decreased incidence in younger and older populations [1, 2]. Meta-analysis has found no predominance for either gender [1]. The estimated minimum annual incidence of TGA is 3.4 per 100,000, though this is likely to be much higher as some people will not present to the hospital and others will be misdiagnosed [3]. While TGA occurs as a single event for many, estimates of recurrence have ranged from 2.9 to 26.3%, but some studies were retrospective [4–8].

Imaging investigations are used to support the clinical diagnosis of TGA. Diffusion-weighted imaging (DWI) on magnetic resonance imaging (MRI) provides specific, consistent findings of 1–5 mm focal lesion in the hippocampal CA-1 sector, which resolve 7–10 days after onset of TGA, with no long-term structural changes [1, 9, 10]. It is hypothesized that, as these neurons are in locations vital for memory consolidation, small lesions may significantly impair memory function, [10] though any effects on prognosis are unknown. For instance, memory and executive function impairment may objectively last up to five days post-TGA onset, despite patients subjectively reporting normal memory [9, 11]. Other patients might develop long-lasting memory problems, especially those with recurrence [12, 13].

Multiple mechanisms have been proposed for the aetiology of TGA. One hypothesis is that retrograde venous flow leads to venous congestion, possibly due to increased thoracic pressure or jugular valve incompetence, which in turn causes a transient ischemia due to hypoperfusion [1, 3, 14–18]. Support for this comes from the observation that many TGA cases are precipitated by Valsalva-like activities, particularly physical or emotional stressors, which would temporarily cause thoracic-pressure elevation [1, 3, 14–16]. Ischaemia from thromboembolism constitutes a contrasting hypothesis on pathogenesis [13, 19]. Some studies have found TGA patients to have higher vascular risk factors and greater frequency of carotid atherosclerosis, suggesting an atherosclerotic embolic event as a cause [4, 20]. One final hypothesis is that TGA is a type of migrainous aura, occurring due to cortical spreading depression, leading to cellular metabolic stress in vulnerable CA-1 sector neurons [9, 18, 21–24]. Genetics may also contribute to susceptibility, based on observations from limited case studies [15, 25–27].

Risk factors that might predispose to TGA recurrence are speculative. In those with recurrence, the presence of multiple risk factors may increase susceptibility. These include continued physical and emotional stressors, arterial hypertension and jugular vein incompetence [1, 2, 4, 6].

To date, TGA research has focused largely on aetiology and pathophysiology, yet our understanding remains incomplete. Few studies have focused on management, prognosis or clinical improvements that can be made to assist healthcare staff in identifying TGA patients more accurately. By identifying patients with recurrence of TGA and comparing them with patients who only experienced a single episode, this study aimed to establish possible risk factors for TGA and its recurrence, so that these patients can recognized and educated on first presentation, with the goal of improving prognostic outcomes and minimising recurrence.

## Methods

### Subjects

Between 2004 and mid-2016, 107 patients presenting with acute memory dysfunction to Joondalup Health Campus Emergency Department (ED) or a neurology outpatient clinic were prospectively screened and recruited into the study; patients having their first attack between 2004 and mid-2016. The supervising neurologist was responsible for making the clinical diagnosis, based on previously published definitions of TGA [2].

### Initial assessment

All patients provided written informed consent to participate in this research. They were interviewed, and the following information was collected: demographic data; details of the episode (duration, symptoms, prior activity); past medical history; family history; and smoking history. A series of investigations were performed after each episode, including MRI with DWI. Consent was obtained from each participant to store results in a secure database. Additional information was obtained from the patients’ ED admission, in instances when they had presented to the hospital.

### Follow-up

**Fig. 1:**
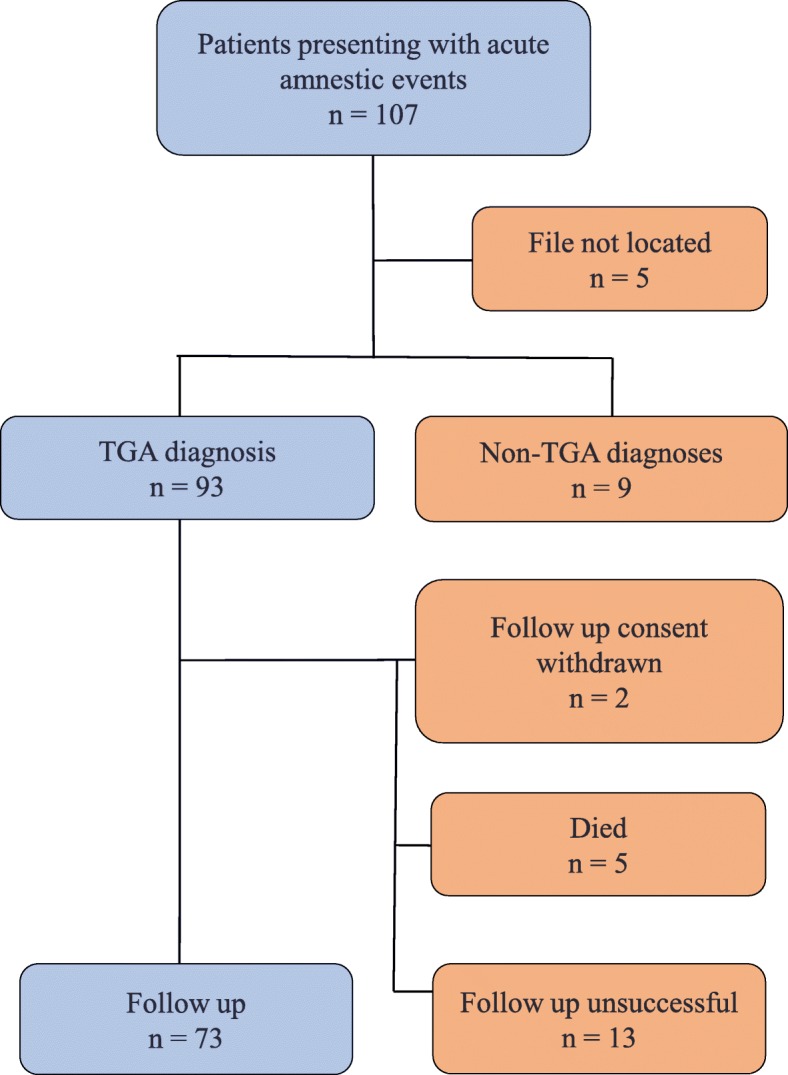
Patient recruitment

In July 2015, the follow-up period commenced. Patients were contacted by telephone to check if there had been any recurrence of an acute amnestic state, to collect any information not gathered from their files and to determine if they had experienced any deterioration in memory. Numbers achieved in follow-up are shown in Fig. 1.

### Statistical analysis

In comparing data groups, descriptive analyses, namely chi-square or Fisher’s exact test with hypergeometric distribution, were used to test the distribution of a factor. Statistical significance was determined at *p &lt;* 0.05.

## Results

### History of presentation

#### Total study population

In a cohort of 102 patients presenting with a transient amnestic state, 93 had experienced one or more episodes of TGA. Mean age of TGA onset was 59.5 (SD 10.3, range 17–78) years, among 49 men and 44 women. In total, these 93 people experienced 117 episodes of clinically diagnosed TGA and 18 recurrent episodes of amnesia, not otherwise specified. Median duration of TGA was 4.5 (IQR: 2–6.5) hours.

In 85 (73%) of episodes, there was an identifiable precipitating event, including: stress (36%); exercise (18%); housework (17%); feeling unwell, often associated with nausea or vomiting (16%); sexual intercourse (8%); hot shower (6%), coughing episode (5%); swimming in cold water (3%); and exposure to chemicals such as fresh paint (2%). Headaches were present either just prior to or during 21% of episodes.

Of the clinical features, repetitive questioning (88%) and disorientation to the day’s events (81%) were most commonly reported. Patients also displayed disorientation to place (54%), day (30%), time (28%), date (26%), and confusion (47%). Anxiety was less commonly reported (3%). No abnormal neurological signs were elicited during the episodes.

**Fig. 2:**
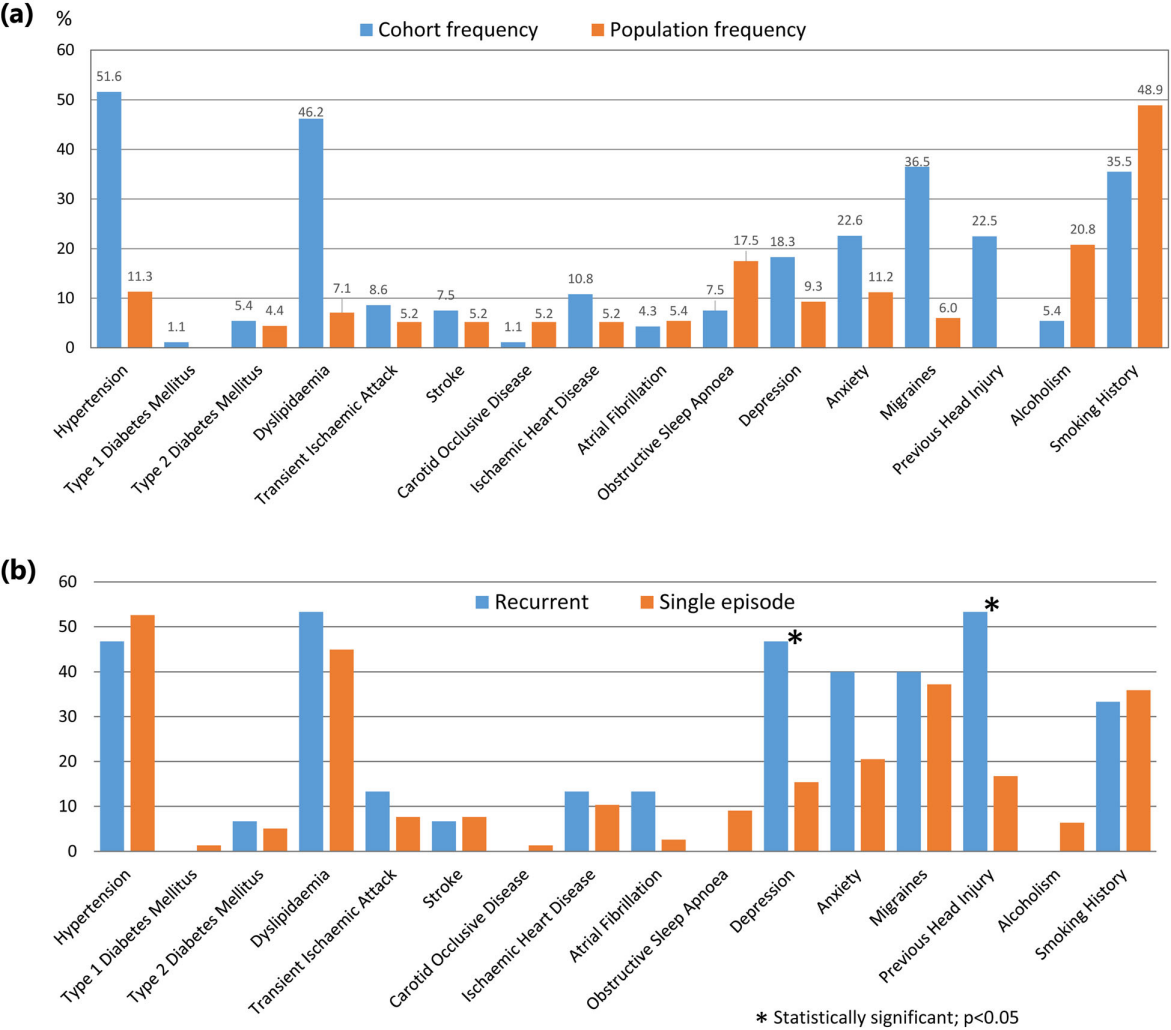
Frequencies (%) of comorbidities: **a** in patients compared to the general population (Australian Bureau of Statistics [29]); and **b** in patients with recurrent (*n* = 15) versus single-episode transient global amnesia (*n* = 78) *p*-values were calculated using the hypergeometric distribution of Fisher’s exact test

When compared as percentage prevalence values, a number of vascular and other risk factors were higher among the TGA cohort than the general population, as described by Australian Bureau of Statistic’s data [28]. These included hypertension, type 1 and 2 diabetes mellitus, dyslipidaemia, transient ischaemic attack, stroke, ischaemic heart disease, depression, anxiety and migraine (Fig. 2a). The Australia Bureau of Statistics did not offer prevalence of these comorbidities by age, therefore our results were compared with the population as a whole.

In total, three (3.2%) patients were later diagnosed with dementia of the Alzheimer’s type, based on the clinical, syndrome functional decline and neuropsychology. Twenty-four (25.8%) had subjective memory deficits only, after neuropsychological testing.

#### Recurrent episodes

Among the 93 patients with TGA, 15 (16%) had recurrent TGA, with 39 episodes in total. The demographics of this group, and the duration of their TGA episodes, were similar to those with solitary TGA (Table 1). Stress (marital status, financial status, relationship, children, work, or a combination) was also the most common precipitating element among those with recurrence. Headache was less frequently noted, with less than a third frequency compared to the isolated event group (isolated vs. recurrent, 27% vs. 8%).

**Table 1:** Patient demographics, history of presenting complaint and past medical history for patients with a single episode of TGA compared to those with recurrent attacks

	Isolated episode		Recurrent episodes		
Patient number	78		15		
Age of Onset (mean)	59.56		59.47		
Age of Onset (median)	61		62		
Age of Onset (Std Dev)	10.83		7.34		
Minimum - Maximum Age	17–78		40–73		
Gender (M)	42	53.85%	7	46.67%	
*Episode characteristics*	No.	%	No.	%	*p*-value
Length of episode (hours +/− Std Dev)	6.14 ± 8.66		5.64 ± 5.66		
Repetitive Question	66	84.62	37	94.87	0.14
Disoriented date	24	30.77	6	15.38	0.07
Disoriented day	26	33.33	9	23.08	0.25
Disoriented time	27	34.62	6	15.38	**0.03**
Disoriented place	41	52.56	22	56.41	0.69
Disoriented day’s events	70	89.74	25	64.10	**&lt; 0.01**
Confusion	40	51.28	15	38.46	0.19
Anxiety	1	1.28	3	7.69	0.11
Headache	21	26.92	3	7.69	**0.02**
*Precipitating event*					
Feeling unwell	13	16.67	6	15.38	0.86
Stress	24	30.77	17	43.59	0.17
Swimming	0	0	3	7.69	**0.03**
Hot shower	7	8.97	0	0	0.09
Coughing fit	2	2.56	3	7.69	0.33
Sexual intercourse	8	10.26	1	2.56	0.26
Housework/gardening	15	19.23	5	12.82	0.39
Gym / Exercise	14	17.95	5	12.82	0.48
Chemicals	4	5.13	0	0	0.30

Regarding clinical features, disorientation to time, disorientation to the day’s events and headache were more commonly documented as having occurred in the isolated-episode group (Table 1).

A history of depression and previous head injury were significantly higher in the multiple-TGA group, compared to the single episode group (Fig. 2b). Recurrent TGA was associated with a family history of dementia (Table 2). Neither family history of TGA nor cardiovascular disease were associated with recurrent TGA. Within our cohort, family history of TGA existed for five patients through a first degree relative, one patient through a cousin, and another patient through two first degree relatives and a cousin. The latter patient also had recurrent TGA; the other six had a single episode.

**Table 2:** Family history in patients with TGA

	TGA once only		Multiple TGA		*p* value
	No.	%	No.	%	
Family history TGA					
No	72	92.3	14	93.3	
Yes	6	7.7	1	6.7	*p* = 0.41
Family history dementia					
No	62	79.5	8	53.3	
Yes	16	20.5	7	46.7	***p*** **= 0.03**

The *p* value was calculated with hypergeometric distribution of Fisher’s exact test

Of the 15 with recurrent TGA, two (5.1%) were later diagnosed with dementia of the Alzheimer type and four (10.3%) reported subjective memory deficits. Among the 78 with an isolated episode, one (1.3%) was later diagnosed with dementia of the Alzheimer type and 20 (25.6%) reported subjective memory deficits.

### Neuroimaging

#### Total study population

MRI studies were performed 120 times, with patients receiving multiple investigations after an episode. Eight (6.7%) were performed within 24 h, 50 (41.2%) were performed 1–10 days after the episode, and 62 (51.7%) were performed after 10 days had passed. Median time between presentation and MRI was 4 days (IQR: 2–24). 24 of 93 patients had DWI spots, 50% of whom had a positive smoking history (*p* = 0.08) and 42% had hypertension (*p* = 0.05). 25 of 26 positive DWIs were taken within 10 days of the episode (96.2%; *p* &lt; 0.0001), with only one (3.8%) positive DWI occurring outside this time, at day 12. In 21 scans, a single spot was seen; two spots in four scans; and three spots in two scans. Average size of spots was 3.5 ± 1.6 mm (mean ± standard deviation). All were in the hippocampus, though the specific location varied anywhere from the hippocampal head to tail; 12 were left-sided, 24 were right-sided. DWI spots had no greater frequency in patients who went on to develop recurrence, dementia or subjective memory problems (Table 3).

**Table 3:** MRI results within 15 days of symptom onset, compared with outcomes in TGA patients

	Normal MRI	Any DWI Spots	Small vessel ischaemic change
Single Event (*n* = 42)	14 [33%]	19 [45%]	22 [52%]
Recurrent episodes (*n* = 15)	5 [33%]	5 [33%]	6 [40%]
Memory Problems (*n* = 16)	6 [38%]	5 [31%]	3 [19%]
Dementia (*n* = 2)	1 [50%]	1 [50%]	1 [50%]

White matter hyperintensities; compatible small vessel; ischaemic change

#### Recurrent episodes

One patient had recurrence of lesions on DWI with their second episode.

## Discussion

Our results for precipitating events leading to TGA were compatible with findings of others [1–4, 14, 25]. Most of our TGA episodes were precipitated by an identifiable event, most commonly an emotional or physical stressor involving Valsalva-like manoeuvres. Some emotional stressors occurred immediately before the attack; others were more longer-term, including home, family and workplace conflicts – it has been suggested that these increase susceptibility to TGA and its recurrence [1, 30]. A history of depression and head injury were significantly increased among those with recurrence.

Approximately one-third of our cohort had a history of migraine; a much greater proportion than in the general population. Our data supports a pathophysiological association between migraine and TGA, probably through spreading depression [15, 21, 24, 27, 28]. Interestingly, one-fifth of our patients had attacks of TGA with headaches.

Other studies have found increased rates of cerebrovascular risk factors in TGA patients [5, 29]; a finding that has not been replicated [2, 6]. Some observers have found increased incidence of ischaemic heart disease in recurrent TGA [4, 20]. However, we did not observe any association with cerebrovascular risk factors.

Incidence of TGA is low, so in cases where multiple family members are affected, it raises questions of whether there is a direct or indirect genetic component. The small sample size of patients with family history of TGA meant statistical analysis was not possible. The presence of TGA in families is not well studied. We have found a complex presentation of TGA in families which requires more investigation. Of note, a family history was found to be significantly associated with the occurrence of TGA, a finding not previously identified.

Family history of dementia was found to be associated with recurrence of TGA. The genetic predisposition of TGA is unknown; however, it is recognised that *APOE e4* is a risk factor for dementia [31]. Therefore, we propose that the link between genetic factors operable in TGA and its relationship to dementia family history may be through an *APOE e4-*like mechanism*.*

Within our patient population, 28% had a second episode of amnesia, though in only 16% was the documentation strong enough to conclusively support that the patient had two or more episodes of TGA within the study’s duration. The reason for this discrepancy is that, while patients and their family members could recall another episode of amnesia, they could not remember other details. Recall bias might have influenced their recollection of the facts.

Another hypothesis for TGA pathogenesis relates to susceptibility of blood vessels around the CA-1 region of the hippocampus, based on observations of DWI lesions in this area [9, 18]. Our findings are not consistent with this hypothesis, with the 35 DWI spots observed located throughout the hippocampus, from head to tail.

All but one spot was detected within one week of TGA onset. We were not able to assess the minimum MRI latency for positive lesions; however, infarctions on MRI within 24 h may only have 82% sensitivity, creating a high frequency of false-negatives in this time frame [32–34]. Repeat MRIs, when performed after one week, showed most lesions had disappeared, in keeping with previous findings. Sensitivity of DWI for acute infarctions after 24 h ranges from 88 to 100%, and specificity is 86-–100% [35, 36].

Factors noted to increase incidence of positive DWIs include a history of smoking and systolic blood pressure ≥ 140 or diastolic blood pressure ≥ 90 mmHg [33]. In our patient population, 50 and 42% of the patients with positive DWI had a history of smoking or hypertension, respectively.

Nevertheless, among TGA patients with MRIs performed within 10 days from onset, 41% (25/61) had a visible, hyperintense lesion. False-negative DWIs may explain why some patients do not show areas of hyperintensity.

Posterior circulation infarctions are much likelier to give false-negative DWIs than an anterior circulation infarction [34]. Blood supply to the hippocampus is complicated as it is variable. In most the posterior cerebral artery (PCA), or a branch thereof, supplies the hippocampus. In some there are contributions from the anterior choroidal artery [37]. The anterior hippocampal artery, branching from the PCA, supplies the hippocampal head, whilst the middle and posterior hippocampal arteries supply the hippocampal body and tail, and have numerous anastomoses [38]. Therefore, small infarctions in the body or tail of the hippocampus might not show DWI positivity because of these anastomoses.

Previous studies have suggested recurrence might be associated with longer-term changes to memory, ranging from verbal and non-verbal memory impairment to dementia [12, 13]. Our data do not support that TGA is a risk factor for dementia. Among the group of people who reported subjective memory impairment, anxiety from the experience of TGA may leave patients doubting their short-term memory capabilities.

## Conclusions

We believe TGA is a distinctive clinical syndrome arising from different mechanisms. It may recur in 16% of those who have a background of head injury, depression or a family history of dementia.

## Data Availability

The dataset supporting the conclusions of this article is electronically stored and is password protected. The de-identified dataset used during the current study is available from the corresponding author on reasonable request.

## Abbreviations

DWI: Diffusion-weighted imaging
ED: Emergency department
IQR: Interquartile range
MRI: Magnetic resonance imaging
TGA: Transient global amnesia
